# What’s New in Addiction Prevention in Young People: A Literature Review of the Last Years of Research

**DOI:** 10.3389/fpsyg.2017.01131

**Published:** 2017-07-06

**Authors:** Cédric Kempf, Pierre-Michel Llorca, Frank Pizon, Georges Brousse, Valentin Flaudias

**Affiliations:** ^1^Laboratoire HESPER EA7425, Université de Lyon–Université Claude Bernard Lyon 1Villeurbanne, France; ^2^EA NPsy-Sydo, Université Clermont Auvergne–Université d’AuvergneClermont-Ferrand, France; ^3^CHU Clermont-Ferrand, Pôle Psychiatrie BClermont-Ferrand, France; ^4^Université Clermont Auvergne – CNRS/SIGMA/Institut Pascal – UMR 6602 – TGI/PEPRADE – ESPE Clermont-AuvergneClermont-Ferrand, France

**Keywords:** health promotion, addiction prevention, young people, child

## Abstract

**Rationale:** Addiction prevention is a major public health problem, particularly concerning young people. Despite the consensus that primary prevention is essential, the evaluation of its impact is questioned.

**Objective:** The objective of this paper is to assess the latest knowledge of addiction prevention programs for young people.

**Method:** Review a collection of research articles using a keyword-based search on three databases: Pubmed, Eric, and PsycInfo. The research was carried out using three groups of keywords and the eligibility study was completed using two criteria: articles published between 2010 and 2017, and articles in refereed journals.

**Results:** Of a total of 13,720 articles in the three databases, 32 studies were included in the review and listed in a grid with five themes: authors, type of population, total population, addictive behavior, and results. Four categories were created based on the objective of the studies: the evaluation of prevention strategies, the study of risk factors for consumption, the prevalence study and other subjects studied. The analysis of the corpus was used to establish a list of risk factors to be taken into consideration in future interventions and research. A list of key elements for performing effective interventions and future research is also proposed.

**Conclusion:** The understanding of the prevention strategies implementation process is discussed as a central element for future research, which will combine stakeholders and researchers. The complexity of the situations and the multifactorial aspects of addiction prevention in young people require a multidisciplinary approach involving the various stakeholders and researchers.

## Introduction

The consumption of psychoactive substances is a world public health concern. Indeed, on average, 6.2 l of pure alcohol is consumed per person worldwide and alcohol consumption is responsible for 35 deaths per 100,000 people (United Nations Office on Drugs, and Crime, 2015). Four deaths per 100,000 people are attributable to illicit drug use and it is estimated that illicit drugs are consumed between 172 and 250 million people worldwide. With regard to tobacco consumption, 21% of adults globally are current smokers (950 million men and 177 million women, World Health Organization, 2015) and tobacco is responsible for 6 million deaths per year (WHO, 2011).

In this context, one of the strategies to control and modify the effects of substance abuse on populations is to use the logic of primary prevention in order to intervene in a concerted way in cases of children and adolescents who are considered at risk. Indeed, it is known that a high consumption of psychoactive substances, particularly alcohol in adolescence, significantly increases the risk of dependence in adulthood (Duncan et al., 1997), and intervention at the age of experimentation or before is seen as important (Cuijpers, 2002; United Nations, Office on Drugs, and Crime (Éd.), 2006). Despite the consensus that primary prevention is essential, the evaluation of its impact is questioned.

A recent review of the Cochrane database (Faggiano et al., 2014) examined school-based programs for illicit drug prevention, which included 51 studies with 127,146 participants. These programs show that there are some discordant results (e.g., high effect versus moderate effect). It is difficult to draw clear conclusions from the heterogeneity of measures and the varied quality of the reported data. The lack of clear conclusions is also a result of the variety of groups targeted by the programs (children, care professionals, stakeholders, etc.), the differences in their aims (group participation, school programs, etc.) and their approach (educational, economic, etc.). Furthermore, this review highlighted the small number of innovative programs that seem to be more effective.

The aim of this paper is to carry out a review of new data available on addiction prevention programs with a particular focus on young people. In spite of the varied methods of evaluation, this review aims to describe the recent development of efficient programs. The conclusions will enable us to make recommendations for future interventions and carry out research on the latest data.

## Materials and Methods

### Search Strategies

A literature search was conducted on three scientific databases: Pubmed, Psycinfo, and Eric.

These three databases were selected in order to cover a wide spectrum of articles, which may also fall within the scope of health sciences, humanities, social sciences, and education. Indeed, addiction prevention among young people can involve one or more of these fields because of the specificity of the target group and the contexts in which the studies have to be carried out (school, family, and other childcare settings, for example).

The choice of these three databases also operates in reference to the biopsychosocial model, (Engel, 1977, 1980; Hoffman and Driscoll, 2000; Frankel et al., 2003; Alonso, 2004; Borrell-Carrió et al., 2004) which attributes disease outcomes to several factors, such as an individual’s physical, psychological, or physiological resources as well as their social and educational environment or interpersonal relationships (Shannon, 1989). We entered three groups of interdependent keywords into each of the three databases as shown below:

**(1)** “Addiction” or “addictive behavior” or “dependence” or “substance abuse” or “substance misuse.” **(2)** “Child” or “school” or “children” or “pupil” or “student” or “teenager.” **(3)** “Prevention” or “health prevention” or “health promotion” or “intervention” or “program” or “education” or “health education.”

The research was conducted on January 30th, 2017.

### Eligibility Criteria

The first eligibility criterion for the articles deals with the period of publication. This review is focused on young people and recent advances in prevention methods, so we determined that the selected items should date from 2010 onward. This permitted us to include longitudinal studies on the specified age group and also allowed us to collect data on the older adolescent age range. The second criterion of publication was that only scientific journals that had been peer-reviewed could be included.

### Study Selection

Having applied the criteria for inclusion, a first researcher (CK) examined the titles and abstracts of articles and excluded those that did not match the three central keywords (children, prevention, addictions).

The items included were all fully read by a researcher (CK) and listed in a grid comprising seven topics: authors, year of publication, target population, addictive behaviors studied, methods, results, and conclusions. A second researcher (VF) read studies that were not clearly excluded, and the final decision on which items to include was made by the authors of the review through discussion.

## Results

### Paper Selection

**FIGURE 1:**
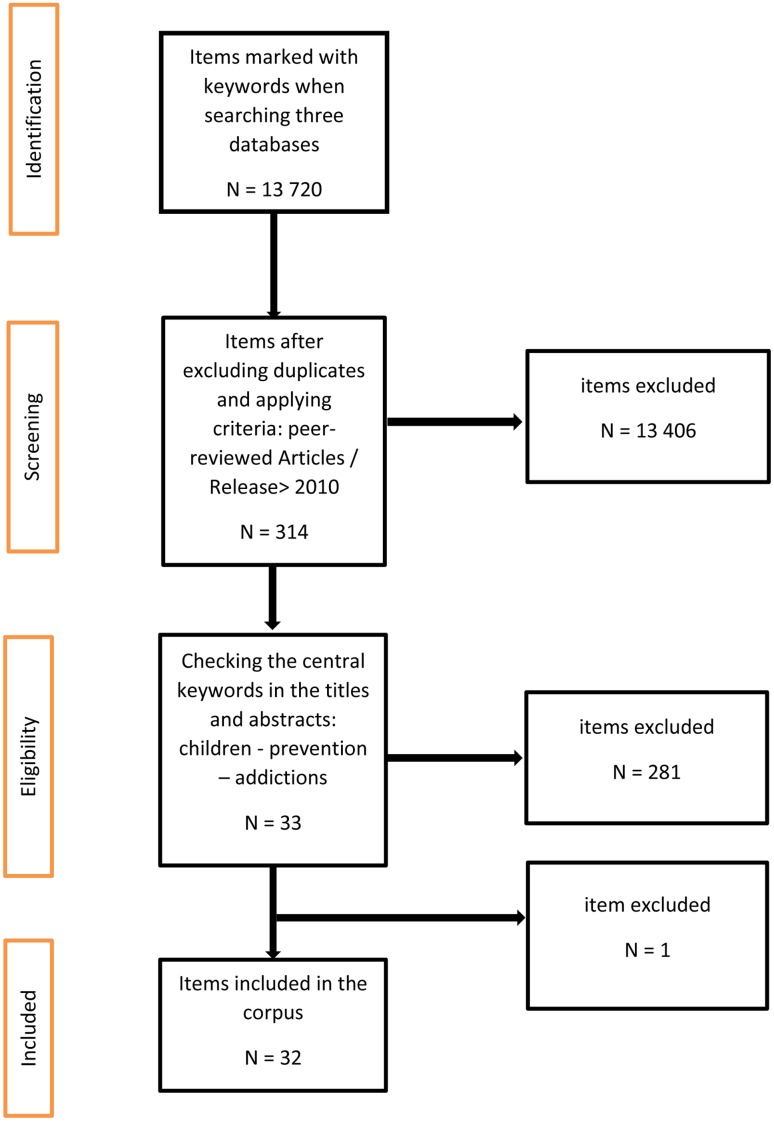
Article review flow chart.

The results of the research identified a total of 13,720 articles in the three databases (**Figure 1**). We then filtered the articles using two criteria: articles in a refereed journal and those published between 2010 and 2017. This reduced the total number of items to 314. A final stage of verification of the titles and summaries of articles was conducted to identify the presence of the central keywords: “children,” “prevention,” and “addictions.” We decided to exclude an article (Juhnke et al., 2013) after the second stage, as the main object of study was bullying in schools, with addictions mentioned as one of the effects of bullying. The corpus was therefore stabilized at 32 articles.

### Global Information about Articles

There are 32 articles discussing studies that are geographically divided between Northern America (15 articles), Europe (11 articles), and Asia (5 articles). The Middle East is represented by one study. Regarding the populations targeted by the studies, children aged 12 are the most represented with 27 occurrences (details available online), then children aged 11 with 26 occurrences. The vast majority of the corpus concerns the age group of children between 10 and 14 years old, and extremes range from 3-year-old children to 30-year-old adults (see **Table 1**).

**Table 1:** Distribution of occurrences of ages in the corpus.

Age	6	7	8	9	10	11	12	13	14	15	16	17 and +
Occurrences	5	6	9	9	17	26	27	20	17	13	11	7


Concerning addictive behavior, half of the corpus (15 items) recounts studies that focus on a single type of dependence. In this half of the corpus, the most represented mono-substance is alcohol, which is mentioned in five articles. Throughout the corpus, alcohol is studied in 16 articles, followed by tobacco in 14 articles and cannabis in 8 articles. Behavioral addiction such as the Internet, video games, and smartphones are present in five articles. Other substances have poorer representation with only two to three mentions, such as cocaine and heroin, prescription medication and inhalants. Finally, it can be noted that eight articles discuss studies that involve more than two substances. The studies were carried out in a number of different contexts. Twenty-two of them took place in schools, three were carried out in the context of social action, five in a family context, and one was based on data from a national general population study on health and drug use.

Due to the wide variety of articles that make up the corpus, we have grouped the articles into three principal categories: articles exploring the effectiveness of prevention programs, studies exploring risk and protective factors, and prevalence studies. Four other studies explored other subjects and were grouped in a separate chapter. We have chosen to add a chapter devoted to the theories used by programs cited in this review in order to better understand the results observed.

### Assessment of the Effects of Prevention Strategies

It should be noted first and foremost that 9 of the 12 studies in this category were carried out in a randomized controlled trial (see **Table 2**). Cervantes et al. (2011) and Spoth et al. (2013) completed their work using a longitudinal study. Finally, the article of Self et al. (2013) presents an exploratory study.

**Table 2:** Synthesis of prevention programs included in the literature review and their theoretical models.

Author	Type of population (age)	Total population	Addictive behavior	Name of program	Theoretical models
Bröning et al., 2014	10–13 years	288	Non-specific	*SFP 10-14 program*	The ecology of human development model
Castellanos-Ryan et al., 2013	7–13 years	172	Alcohol and other drugs	*MLES program*	Family social learning model
Cervantes et al., 2011	11–14 years	153	Non-specific	*FAMILIA ADELANTE Program*	*N.C.*
Faggiano et al., 2010	12–14 years	7,079	Alcohol, tobacco, cannabis	*UNPLUGGED program*	Comprehensive social influence model Social Learning Theory Social Norms Theory Health Belief Theory (see MMH) Theory of Planned Behavior Theory of Problem Behavior
Mares et al., 2011	11–12 years	656	Alcohol	*IN CONTROL: NO ALCOHOL! Program*	Social Learning Theory
Miovský et al., 2015	11–13 years	1,874	Cannabis	*UNPLUGGED program*	Comprehensive social influence model Social Learning Theory: Social Norms Theory Health Belief Theory (see MMH) Theory of Planned Behavior Theory of Problem Behavior
O’neill et al., 2011	9–11 years	2,512	Alcohol and tobacco	*MMH Program*	Health Belief model
Self et al., 2013	10–12 years	8,721	Non-specific	*Device 4-H*	Theory of Planned Behavior
Spoth et al., 2013	11–12 years	11,960	Alcohol, tobacco, cannabis, inhalants, methamphetamine	*PROSPER Device*	*N.C.*
Valdivieso López et al., 2015	11–15 years	1,583	Tobacco	*Tarragona program*.	*N.C.*
Vermeulen-Smit et al., 2014	11–12 years	213	Alcohol	*IN CONTROL: NO ALCOHOL! Program*	Social Learning Theory
Weichold, 2014	10–15 years	1,693	Alcohol and tobacco	*IPSY Program*	Theory of behavioral problems


#### The PROSPER Device

The PROSPER (PROmoting School – community – university Partnerships to Enhance Resilience) device, which was studied in Spoth et al. (2013) and developed by the Prevention Science Institute at the University of the State of Iowa, is not a program dedicated to addiction prevention. This is primarily a device that allows the construction of a partnership between the school, the University (part of the American national network Cooperative Extension Systems) and local stakeholders in order to find effective answers on the subject of addictions and problematic behaviors in young people. Therefore, the aim of this partnership is to determine the most relevant intervention programs with regard to the context. In the article by Spoth et al. (2013), the PROSPER device has enabled the different partners to select adequate prevention programs in order to study a cohort over 6.5 years. Four programs were chosen: the “Strengthening Families” program (SFP 10-14, described below), the “Life Skills Training” (LST), the “Alert,” and the “All Stars” programs.

The *LST program* (Botvin and Kantor, 2000) is an intervention that is designed to promote the development of social skills, such as strategies for avoiding the use of substances. *Project ALERT* focuses on social influence (Rosenstock et al., 1988) and works on substance abuse norms, focusing on peer and media pressure.

The *All Stars Program* is an intervention which works on students’ abilities to avoid the negative behavior induced by substances (Hansen, 1996).

These four programs, scientifically validated by the National PROSPER Network (Iowa State University, 2015) were carried out over 2 years on young people from ages 11 to 13 and their parents. Each chosen cycle had several group sessions (7 for “SFP 10-14,” 15 for “LST,” 11 for “Alert,” and 13 for “All Stars”) which mainly took place during school time and its objective was to develop parental skills as well as young people’s intra and interpersonal skills. The stakeholders were school teachers and social workers who intervene locally with families and young people.

The Illicit Substance Use Index (Spoth et al., 2001) was used in order to assess PROSPER’s operative effect on substance consumption among young people. The index revealed an 18.8% reduction in the consumption of five substances (alcohol, tobacco, inhalants, methamphetamine, and cannabis) for the intervention group, 5 years after the intervention. This reduction was then at 15%, 6 years after the end of the intervention.

#### The “4-H Health Rocks” Device

Just like PROSPER, the “4-H Health Rocks” studied in the article of Self et al. (2013) is not, in itself, an addiction prevention program. It is a global model of multi-thematic intervention based on a territorial and community intervention which promotes the collaborative work between young people and adults. This American national device from the University of Minnesota is controlled by the “Minnesota 4-H Foundation.” Self et al. (2013) presents the results of this device’s intervention on the promotion of the health of young people (aged 8–14) and community education about drugs, alcohol, and tobacco in the context of holiday camps in its study. This intervention includes a 10-h program of interactions between young people and adults. The intervention aims to develop life skills and was evaluated by a questionnaire featuring 36 questions grouped around four concepts (actions, knowledge, beliefs/attitudes, and life skills) at the start of the intervention and again at the end.

All comparisons, pre and post measurement, showed a significant difference. Thus, all scores after the intervention were higher than those achieved upstream, which tends to confirm the positive effect of the intervention on participants. Indeed, 96% of participants had an increase in knowledge of the risks and consequences associated with tobacco consumption, 95% of participants improved their social skills (e.g., social and/or resistance), and 96% of participants disapproved of tobacco products.

#### Program: “Unplugged”

The studies of Faggiano et al. (2010) and Miovský et al. (2015) refer to the European program “Unplugged.” This program was designed to be used by teachers, who had previously been trained, within 1 or 2 school lessons. It was comprised of 12 sessions which had the same structure: a time schedule, a rationale (what the lesson is about, why it is important, what evidence there is), objectives, and a list of materials needed (European Monitoring Centre for Drugs and Drug Addiction [EMCDDA], 2015b).

The study by Miovský et al. (2015), however, refers to “Unplugged” to specifically investigate its effect on the use of cannabis by young Czechs. This study involved 1,874 teenagers put into three groups based on their probable level of risk of consuming cannabis (low risk, moderate risk, and high risk). In each of these groups the subjects were placed randomly in an intervention group and a control group. Significant differences were shown between the control and intervention groups in each of the groups considered at risk. The probability of cannabis usage in the ‘high risk’ group was 25.51% for the intervention group and 32.61% for the control group. For the ‘low risk’ group, this probability was 4.3% for the intervention group and 6.53% for the control group. School results, failure to comply with the rules and suicidal thoughts were the three most important risk factors which differentiated the ‘low risk’ and ‘high risk’ groups.

In the work by Faggiano et al. (2010), the “Unplugged” program is used with young people between 12 and 14 years old, as well as their parents, for a total of 12 group sessions of 45 min. The teachers receive 12 h of training before the sessions. The sessions are divided into three parts: the first provides a work-around knowledge of attitudes toward addictive behavior, the second part focuses on the relationship issues between peers, and the last part deals with the development of interpersonal skills. A questionnaire is used at the beginning of the program, then 6 and 18 months after, and includes 37 items. The items in the questionnaire are derived from the Evaluation Instrument Bank of the European Monitoring Centre for Drugs and Drug Addiction (see European Monitoring Centre for Drugs and Drug Addiction [EMCDDA], 2015a for more information). The results obtained in the study showed persistent effects 18 months after the intervention. The prevalence of alcoholic intoxication episodes was 10.5% for the control group and 8.3% for the intervention group. For cannabis, it was 5.5% for the control group and 3.3% for the intervention group.

#### Program: “In Control: No Alcohol!”

Mares et al. (2011) and Vermeulen-Smit et al. (2014) studied the Dutch program “In Control: No. Alcohol!” which is based on another American prevention program “Smoke Free Kids” and has been implemented in the Netherlands. This program caters to young people and their mothers. The use of online tools allows the families to engage in the project while avoiding the constraints of a complex program which often consumes time and requires frequent travel. “In Control: No. Alcohol!” consists of five sessions during which activities to be carried out at home are suggested (games, quizzes, puzzles, information, etc.). Each session lasts 1 month and has a main theme: Session 1: general information about alcohol and the importance of parental support; Session 2: the risks of alcohol for young people; Session 3: parental attitudes toward alcohol and rules; Session 4: the influence of peer pressure; Session 5: the influence of the media and respect for the rules. An online questionnaire is used to carry out the evaluation of the program; there is one questionnaire for mothers and one for the young people. This questionnaire is used 6 months after the end of the program and again after 12 and 18 months.

Vermeulen-Smit et al. (2014) showed that the results of the program, which sought to show the effects of parental communication methods and behavior (intensity and frequency of mother–child communication, rules concerning alcohol, drinking agreements, parental control outputs of the child) on their children (negative perception of alcohol consumption, intention to consume alcohol). The evaluations were carried out 5 months (T1) and 12 months (T2) after the intervention. After T1, the intervention had significantly increased parental behaviors regarding the frequency of alcohol-specific communication and the agreement of non-consumption and control outputs. These results had unexpected effects on T2 concerning the negative perception of alcohol; an agreement on alcohol between parents and children led children to have a negative perception of alcohol. Furthermore, in T2 the frequency of parent–child communication increased this negative perception. The quality of communication between young people and parents, along with the specific rules concerning alcohol, decreased the young people’s intentions to consume alcohol.

#### Program: “SFP 10-14”

The study of Bröning et al. (2014) examined the German adaptation of the “Strengthening Families Program: For Parents and Youth 10-14” (SFP 10-14), an American program that was derived from the work of Iowa State University and was first carried out in 1993.

SFP 10-14 is aimed at parents and children. It consists of 7 weeks of thematic interventions followed by a 4-week reinforcement program 4 to 6 months afterward. Three stakeholders, who may be experienced social workers, lead interventions with groups of 8 to 12 families. Specific sessions for children are organized around topics such as stress management, peer pressure and influence, as well as the feeling of self-efficacy. For parents, the program incorporates sessions on the development of parental support. Other sessions bring together parents and young people to engage in intra-family communication.

The evaluation of the program was organized into four sections: the beginning of the intervention (t0), the end (t1), 6 months later (t2) and 18 months later (t3). Several methods were used at t0 and t3 and concerned children and their parents (11 different questionnaires and urinalysis specifically for the children). The results of these measurements are not available for the moment. However, the implementation conditions (intervention and the scientific assessment device) were seen as positive by parents, their children and stakeholders, who indicated that it was a good adaptation of the SFP 10-14 program.

#### Program: “IPSY”

Weichold’s study (Weichold, 2014) presented a German program designed at the University of Jena entitled “Information + Psychosocial skill = Protection” (IPSY) and based on the skills approach initiated by the World Organization of Health (WHO-Department of Mental Health, 1999). It aims to promote young people’s psychosocial skills from 11 to 13 years old as a way of protecting against the consumption of addictive substances. A total of 33 h of intervention are organized over a period of 2 years. At the age of 11, interventions are focused on the development of intra and interpersonal skills. At 12 and 13 years old the program aims to strengthen these skills. This is implemented by teachers who have already been trained. Interventions by older children are also organized to encourage the creation of a positive influence among peers.

A pilot study showed that this program is well-liked and has been found to be practicable for teachers (Weichold and Silbereisen, 2012). A set of studies explored the efficacy of the program, particularly the longitudinal study. Two groups of children were observed: one group who received the intervention for 3 years, and another who had no intervention. The evaluation showed a decrease in prevalence, frequency and amount of alcohol and cigarettes used for the intervention group (Weichold and Silbereisen, 2009). This group was also shown to have improved general life skills, competences and knowledge about substances. The program had a greater impact on women (Weichold et al., 2012), and an even greater impact on people without problematic usage (Spaeth et al., 2010).

#### Program: “Familia Adelante”

The particularity of the American program “Familia Adelante,” presented in the study of Cervantes et al. (2011), is that language factors are taken into account in matters of prevention. The “Familia Adelante” program is based on the Hispanic Family Intervention Program of the National Institute of Mental Health (NIMH) and the Spanish Speaking Mental Health Research Center at the University of California in Los Angeles.

The program mainly consists of interventions based on coping methods, (Paulhan, 1992; Park and Folkman, 1997; Matthieu and Ivanoff, 2006) which help with the management of stressful situations and the development of skills. Paulhan (1992) recalls the definition of coping: according to Lazarus and Folkman (1984) it is “the set of cognitive and behavioral efforts to master, reduce or tolerate internal and external requirements that threaten or exceed resources of an individual.” The intervention is organized with young people and their parents in 12 group sessions (groups of 8 to 10 subjects) of 90 min outside of school time. Each session is accompanied by a guide which clarifies the objectives, activities, and the necessary equipment.

The program is evaluated by interviews and psychometric scales (e.g., Hispanic Stress Inventory; Conners Children’s Behavioral Parent Rating Scale). The measures are organized at the first meeting, and at the latest 6 months after.

The results show that the majority of the themes have important efficiency scores. For parents, the highest scores relate to the risk of human immunodeficiency virus (HIV) and the management of relationship problems with their children. The lowest score is their children’s anxiety. For children, the highest scores are the level of communication with their parents and social norms, whereas the use of condoms is their lowest concern. The program has shown significant efficacy in the improvement of communication skills. The effects on the decline in consumption of products are also significant.

#### Program: A Subsample of the “Montreal Longitudinal and Experimental Study” (MLES)

The article by Castellanos-Ryan et al. (2013) presents a Canadian intervention, which is carried out over a period of 2 years with children aged 7–9 years old (started in 1985), and the assessment of its impact 6.5 years after by a longitudinal study (MLES). This program specifically targets young boys and their parents. It targets the development of children’s social skills and parental support.

The program includes sessions of 45 min in small groups of four to seven boys and its objective is to promote healthy relationships between peers. Sessions are conducted by four professionals in alternation (psychologist, social worker, therapists). Sessions for parents are organized in the family home by different professionals who are already involved with the children. The objective of these sessions is the guidance and support of parents in their roles, particularly those involving the monitoring of their sons’ homework and their behavior outside of the house.

The interventions with children are filmed, while those with parents are recorded in audio format. Two questionnaires are available to children at the end of the program: the Self-Reported Antisociality Questionnaire for substance use (LeBlanc and Fréchette, 1989) and Social Behavior Questionnaire for impulsiveness (Tremblay et al., 1991).

The results showed that the intervention led to a reduction in antisocial behavior in young people, decreased impulsiveness, improved parental supervision, increased school engagement as well as a decline in relationships with deviant peers. These results showed a 47% decrease in the frequency of alcohol at 14 years old, and a 50% decrease in the number of drugs consumed between 14 and 17 years old.

#### Program: “Tarragona Tobacco Prevention” Program

The last program in the first category of articles in the corpus is presented in the article of Valdivieso López et al. (2015). It discusses a tobacco prevention program in a Catalan region in Spain (Tarragona). The hypothesis of the study is that preventive interventions that are integrated into the school curriculum will have a greater impact on the decline of the incidence and prevalence of smoking among adolescents if they are conducted by school nurses.

Seven modules, including sessions in classes, workshops, and activities were organized over 4 years (2007–2011). The modules that took place during school time were led by the school nurse in association with teachers. These modules were integrated into the school curriculum (nine sessions over 3 years). Other modules included activities to be carried out at the children’s homes with their parents, and others were dedicated to the organization of festive events.

Results showed no significant differences in the incidence and prevalence of the use of tobacco in spite of a drop of 25 and 26%, respectively. The authors hypothesize that these results are due to not enough contact hours (9 h over 3 years) compared to the 16 sessions over 3 years as suggested by the European Smoking Prevention Framework Approach (de Vries et al., 2003).

#### First Conclusions about the Assessments of Prevention Strategies

The 12 articles observe the positive effects of programs on lowering consumption and experimentation with substances, particularly alcohol and tobacco, among children. All of the assessed programs have several aspects in common. First, the intensity of the intervention over a long period is a constant. Thus, and as indicated by Valdivieso López et al. (2015), a relevant intervention is a procedure that is based on a minimum of 10 sequences over 3 years.

Another aspect common to the programs is the content of the interventions. Indeed, the central theme is the development of the children’s and adolescents’ skills as a protection factor. The building of skills allows children to equip themselves to deal with the life situations they may encounter, with relative autonomy. Risk information or knowledge of substances are important elements in an intervention but cannot be considered as the unique strategy (Medina-Mora, 2005). The development of skills allows children to find appropriate responses to problematic situations (peer pressure and influence, for example) and gives them the opportunity to build experience that increases with age. O’neill et al. (2011) consider that it is the most powerful protection factor.

The final joint aspect of the 12 papers is that the interventions aim to act on the environment of the children with the participation of those close to them, such as parents, teachers, adults, or social workers. Vermeulen-Smit et al. (2014) specifies, for example, that parents who improve their intervention skills have positive effects on lowering their children’s alcohol usage. Moreover, the majority of the programs cited above are largely constructed on social and cognitive psychology theories.

### Theoretical Models Used in Prevention Programs

Of the 12 articles found in the previous category, eight specify the theoretical foundations that were used in the development of the prevention programs studied (see **Table 3**).

**Table 3:** Synthesis of articles related to the study of factors associated with consumption.

Author	Type of population (age)	Total population	Addictive behavior	Results	Factors associated with consumption (risks or protective)
Barnard and Bain, 2015	3–14 years	6	Non-specific	The support of parent drug users through social interventions to reduce the risk of consumption in their children.	- (Risk) Low support from adults in educational community - (Protective) Support and communication skills of parent and social workers
Dickens et al., 2012	11–19 years	2,582	Alcohol	The support of the educational community and the influence of peers are two factors that have a positive effect on the reduction of alcohol consumption in under-16s.	- (Risk) Low support from adults in educational community - (Risk) Weak attachment to school - (Risk) Influence of peers - (Protective) Support and communication skills of parent and social workers
Emory et al., 2015	10–13 years	688	Tobacco	Prevention messages in youth campaigns have a probability of reducing testing by 30%.	- (Protective) Prevention campaigns
Farmer and Hanratty, 2012	10–15 years	3,903	Tobacco, alcohol, illegal drugs	Boys face overall higher consumption than girls. The feeling of well-being is a protective factor. The consumption of substances varies with age.	- (Risk) Gender: being a boy
Fernandez-Hermida et al., 2013	11–19 years	7,065	Alcohol	The 79.1% of parents underestimate their children’s alcohol consumption. Parents are mostly convinced that their children do not have access to drugs.	- (Risk) Underestimation of parents of their child’s consumption
Gaffar et al., 2013	10–21 years	3,923	Tobacco	Low academic achievement, friends’ consumption of tobacco and khat, being a boy, having a sense of high stress and the amount of pocket money are factors associated with the consumption of tobacco and khat.	- (Risk) Low educational outcomes - (Risk) Influence of peers - (Risk) Gender: being a boy - (Protective) Support and communication skills of parent and social workers
Liu et al., 2013	6–17 years	605	Cannabis	Situations of abuse in childhood have an effect on the use of cannabis in adolescence.	- (Risk) Situations of aggression - (Risk) Gender: being a boy
Marsiglia et al., 2011	ages 10–12	1,473	Alcohol, tobacco, cannabis, inhalants	Linguistic acculturation, particularly through the media, is a factor associated with substance use. Girls are more sensitive to tobacco consumption. Boys are more likely to consume cannabis. Introduction to inhalants is very present in 10 year olds.	- (Risk) Low linguistic acculturation
Ridenour et al., 2012	8–16 years	1,147	Alcohol, tobacco	Situations of chronic stress are predictors for drug use.	- (Risk) Situations of aggression
Traube et al., 2012	>11 years	827	Heroin, crack, cocaine, cannabis, tobacco, alcohol	The family placement of children is not a risk factor for drug use. The intensity of the child’s relationship with social workers’ referents has a positive effect on drinking.	- (Protective) Support and communication skills of parent and social workers
Wang et al., 2013	11–19 years	52,214	Alcohol	A link is shown between the presence of shops selling alcohol and consumption of alcohol by children.	- (Risk) Facility of access to the product
Zorbaz et al., 2015	8–10 years	396	Video games	Family relationships, time spent on the computer and educational outcomes are the most important factors regarding an addiction to video games. Boys are more affected than girls.	- (Risk) Low outcomes - (Risk) Gender: being a boy


Psychology is the disciplinary field in which the theories and program models have been developed. They have their foundations in social psychology and cognitive psychology which fit the theoretical underpinnings of the programs. The major theoretical models:

#### The Ecology of Human Development Model (Bronfenbrenner, 1979)

This theoretical model allows us to consider the situation of an individual through their relationships with various systems. These relationships are more or less direct interactions with the individual. Thus, it is necessary to understand the different systems as a whole (microsystem, mesosystem, exosystem, macrosystem, and chronosystem) in order to accompany individuals.

#### Family Social Learning Model (Patterson, 1975)

People are the agents of behavioral change. Here, it is the family members who have the key to change through mutual learning. The principle of reinforcement plays a central role in this theory.

#### Comprehensive Social Influence Model (Sussman et al., 1995, 2004)

Acting against social pressures can effectively prevent drug use. Similarly, information and training focused on social influence can help limit the idea that drug use is common in a group of peers.

#### Social Learning Theory (Bandura, 1971, 1989)

Changes in an individual’s behavior can be explained by two factors: observation and imitation. The development can be instigated by change, which can be made directly or through an intermediary.

#### Health Belief Model (Rosenstock, 1974, Rosenstock et al., 1988)

This model attempts to explain how the individual’s behavior regarding their health is related to their perceptions, which are influenced by various internal and external factors and are expressed by a risk-benefit balance.

#### Theory of Planned Behavior (Ajzen, 1991)

Motivation drives the will to act. This motivation influences the attitudes and norms of the individual. These same attitudes are determined by individual beliefs that establish positive or negative effects on the behavior concerned. The norms concern the importance that a person places on the opinions of others.

#### Theory of Behavioral Problems (Jessor, 2001)

Behaviors are influenced by the values, beliefs, and attitude of an individual as well as friends and family’s perception of these behaviors. The development of life skills is central to the aim of changing behavior.

These theoretical models seek to understand and act on patterns that cause health problems. The anchoring of their theoretical foundations in social psychology favors the suggestion that health behaviors are the result of complex relationships between the individual, collective, and environmental factors (Rosenstock et al., 1988). We should also note the Theory of Planned Behavior (Ajzen, 1991) shown in the “4-H” device, the theory of behavioral problems (Donovan and Jessor, 1985) in the “IPSY” program and the Health Belief Model (Rosenstock, 1974; Janz and Becker, 1984) in the “MMH” program.

For the programs “UNPLUGGED,” “IN CONTROL: NO ALCOHOL!,” and the Canadian program, the theoretical framework is shared and is based on Albert Bandura’s work on social learning (Bandura, 1971) and more broadly on social cognitive theory (Bandura, 1989). Thus, the concept of skill, developed in the theory of social learning (Bandura, 1971) comes in support of issues relating to the behavior of individuals. Note also that the “UNPLUGGED” program refers to the theory of social influence (Sussman et al., 1995; Dijkstra et al., 1999) and also the Social Learning Theory, the Social Norms Theory, The Health Belief Theory, The Theory of Planned Behavior, and the Theory of Problem Behavior.

Moreover, we can observe that Albert Bandura’s works, especially those on the sense of self-efficacy, are also present in the Theory of Planned Behavior mentioned above for the “4-H” device. This indicates a strong prevalence of socio-cognitivist theory in prevention programs.

Finally, the program “SFP 10-14” refers to the work of the psychologist Urie Bronfenbrenner on the ecology of human development (Bronfenbrenner, 1979), itself present in Social Learning Theory when it comes to building the development of the individual’s skills (Ladd and Mize, 1983).

### Factors Associated with Consumption Studied in the Last Years

This category, which represents 12 articles of the 31 in the corpus, brings together works that have in common the study of a number of environmental risk factors (Ostaszewski and Zimmerman, 2006), in particular, those that can have an impact on children’s and adolescents’ addictive behaviors (see **Table 3**). Two types of environments were highly represented: the school environment and home environment (parental and other). Gender and campaign prevention were two other influencing factors.

#### School Environment Factors

The school environment contributes some influencing factors, such as low educational outcomes (Gaffar et al., 2013; Zorbaz et al., 2015), low support from the adults in the educational community (Dickens et al., 2012; Barnard and Bain, 2015), the weak attachment of children to their school (Dickens et al., 2012), aggressive situations (Ridenour et al., 2012; Liu et al., 2013) and the influence of peers (Dickens et al., 2012; Gaffar et al., 2013).

Indeed, Gaffar et al. (2013) showed that in a large population of intermediate and secondary school students (*N* = 3,923), the major predictive factors for tobacco consumption was academic performance and having friends who used substances. The authors also observed that feeling stressed is also a risk factor. Dickens et al. (2012) showed that peer influence is not only a risk for young people (aged < 16 years), but also for older students when consuming alcohol. As well as the parental environment (living with biological parents, cultural identification, etc.), a weak attachment to school is also an important risk factor. Barnard and Bain (2015) studied children from substance-misusing families and their alcohol consumption. They observed that the educational community (such as social workers) is an important protective factor for vulnerable children.

Liu et al. (2013) showed that intervention projects that work on aggressive behavior reduce these behaviors, as well as the consumption of marijuana. These elements were confirmed by Ridenour et al. (2012) who showed that exposure to violence increases the risk of behavioral problems. These authors also highlighted that the school’s commitment to all these questions is a major factor (in spite of the difficulty for those working on prevention to mobilize the public).

#### Parental and Proximal Environment Factors

The study, which focused on 3,587 parents and 7,065 children between 11 and 19 years in five European countries (Sweden, Slovenia, Czechia, Spain, and Portugal), helped Fernandez-Hermida et al. (2013) show that parents’ underestimation (79.1%) of their children’s access to and consumption of substances, is a risk factor associated with consumption.

Marsiglia et al. (2011) studied the impact of linguistic acculturation on the consumption of substances with 1,473 Latin American students aged 10 to 13. The results support the hypothesis that linguistic acculturation and the consumption of substances have complex relationships among the young Latin Americans living on the border between Mexico and the United States. For young girls, acculturation and media use is associated with a strong uptake in smoking. For young boys, the association shows a marked increase in cannabis consumption.

Wang et al. (2013) investigated the risk posed by the proximity of products with 52,214 Taiwanese students in colleges and high schools. A link is shown between the presence of shops selling alcohol in the first 6 months of their opening, and students’ levels of consumption in their schools. Wang et al. (2013) therefore recommend acting on environments using a community approach.

Finally, other so-called ‘protection factors’ (Epstein et al., 2001) are turned into relationship and communication issues. Some authors (Dickens et al., 2012; Barnard and Bain, 2015) show that the support of parents and their ability to communicate with their children are powerful factors in protection; as are ongoing relationships between social workers, parents, and children (Traube et al., 2012; Barnard and Bain, 2015).

#### Other Factors

Finally, two individual factors, considered as the risk and/or predictive factors, are related to gender and age. Indeed, for many authors (Farmer and Hanratty, 2012; Gaffar et al., 2013; Liu et al., 2013; Zorbaz et al., 2015) boys at the age of adolescence are generally more affected by addictions than girls.

Regarding the effects of global communication in prevention, the study of Emory et al. (2015) estimates that media prevention campaigns reduce tobacco experimentation by 30% for teenagers who are receptive to cigarettes and tobacco control advertising. But this teenage population represents only 30% of their total population (N total = 688).

### Prevalence of Addictive Behavior in Young People

The articles in this category are designed to study the prevalence of certain addictive behaviors. In our corpus, the major prevalence studies of addictive behavior study were about Internet usage (see **Table 4**). They set an addiction prevalence at around 10% for a relatively close age group (7–16 years). The study of Lan and Lee (2013), which focused on 1,045 students, saw that 10.9% of the population is classified as having an addiction to the Internet. The 20.9% of children are prone to depression, which is one of the possible factors that can lead to Internet addiction. The main predictive factors that have been identified for developing an addiction to the Internet are: depression, hours of Internet use on weekends and holidays, and the kind and frequency of Internet usage. Boys show significantly more addictive behaviors toward the Internet than girls. Interpersonal relationship problems and depression are the most problematic symptoms in this age category.

**Table 4:** Synthesis of prevalence studies.

Author	Type of population (age)	Total population	Addictive behavior	Results
Collins et al., 2011	10–17 years	1,105	Drugs, tobacco, alcohol, cannabis	The prevalence of alcohol consumption is 45%. The 35% for prescription medication, 28% for tobacco, and 17% for cannabis.
Lan and Lee, 2013	8–12 years	1,045	Internet	The 10.9% of children have an addiction to the Internet. Boys are more affected than girls. Relationship problems and depression are the most present symptoms.
Li et al., 2014	7–16 years	42 studies	Internet	The prevalence of an addiction to the Internet may vary between 2.5 and 27%. It is estimated that overall prevalence is 10%.
Li et al., 2014	7–16 years	24,013	Internet	The prevalence of Internet addiction is 6.3%. The 11.5% of elementary school students have an addiction.


Li et al. (2014) show that 6.3% of the total sample (24,013 students) have an addiction to the Internet. The 12,993 of the students in the sample use the Internet, and 1,523 of these have an Internet addiction, showing that 11.7% of Internet users have an addiction (1,523/12,993). The results also show that 11.5% of elementary school students have an addiction to the Internet. Students at schools in rural areas are more affected than those at schools in urban areas. The operating time is the main factor associated with an addiction to the Internet. Children in primary schools who use the Internet to play and communicate are more likely to develop an addiction (+22.5% for games and 13.5% for communication).

Collins et al. (2011) show in their study of 1,105 American students, that the prevalence for alcohol consumption is higher than drugs (46% vs. 35%), tobacco (28%), and cannabis (17%). Among children aged 10 to 13, the prevalence of drug consumption exceeds that of alcohol (27% vs. 24%). The most consumed drugs are those used to manage pain (27%), sleep (16%), sedatives (10%), and amphetamines (6%).

### Other Articles

The last group of articles brings together four studies involving very different subjects (see **Table 5**).

**Table 5:** Summary of articles on recommendations of CDC, validation of scale on smartphone addiction, and model of consumption.

Author	Type of population (age)	Total population	Addictive behavior	Results
Dean et al., 2014	10–30 years	55,772	Tobacco, alcohol, cannabis, cocaine, heroin, inhalants, stimulants, hallucinogens, drugs	Construction of six models of consumption. The earlier the initiation, the earlier the risk of regular consumption.
Kim et al., 2014	6–16 years	795	Smartphone	Validation of a scale for the measurement of a Smartphone addiction. Coefficient alpha of Cronbach to 0.88
Inman et al., 2011	3–18 years	Thirty-nine programs (including seven on addictions)	Alcohol, tobacco, other drugs	Reminder of the seven recommendations established by the CDC on tobacco prevention programs. Addiction prevention programs must register more generally in a school’s health promotion strategy.
LeNoue and Riggs, 2016	Not available	Four Principal programs	Alcohol, tobacco, other drugs	There is not enough scientific research to informed about efficacy of prevention interventions.


The study by Kim et al. (2014) confirms the growing interest in research on behavioral addictions since the subject concerns the validation of a diagnostic scale of smartphone addictions among 795 South Korean students. The initial scale was made up of 29 items. The reliability tests for the scale kept only 15 of the items, which were regrouped into four areas: disturbance of adaptive functions, virtual reality orientation, withdrawal, and tolerance. A Cronbach’s alpha of 0.880 indicates that the scale is very reliable.

In their paper, Dean et al. (2015) presented the results of a study of a population of 55,772 individuals from ages 10 to 30, whose goal was to discover different types of drinks “initiations” models through a new approach called the MEPSUM (Multi Event Process SUrvival Mixture) model (Dean et al., 2014). Six models of drug consumption (tobacco, alcohol, marijuana, cocaine, heroin, inhalants, stimulants, hallucinogens, and other drugs) were identified: abstinence, late use of “soft drugs,” early “soft drugs,” progressive use of “soft drugs,” late consumption of “hard drugs,” and early consumption of “hard drugs.” The results determined that the probability of the maximum consumption risk at a given age is dependent on the age of initiation. Therefore, the earlier the initiation, the earlier the risk of regular consumption.

Inman et al. (2011) worked on a review of literature on evidence from 39 health promotion programs at schools, including seven on addictions. The authors recommend that future programs should be based on the socio-ecological health model. Seven recommendations are given for school health programs aimed at the prevention of tobacco usage: develop rules on tobacco consumption; provide information on the negative effects on health and social consequences of short and long term use of tobacco, social influence on the consumption of tobacco and the peer pressure relating to smoking and the skills required to be able to resist consumption; develop preventive actions from kindergarten to high school; develop action for training teachers; involve parents and families in prevention programs; support students and adults in the educational community in their efforts to stop smoking; and evaluate prevention programs at regular intervals.

More recently LeNoue and Riggs (2016) reviewed some programs of prevention and distinguished two types of programs for young people: “school-based prevention” (for example the Unplugged program, the life skills programs and the good behavior game) and “non-schooled-based prevention.” They conclude that there is a gap concerning the efficacy of some programs and their implantation on national public health program.

## Discussion

Our literature review aimed to show recent developments in the prevention of addictive behavior. Our results showed the different risk factors for consumption and the effectiveness of many prevention programs. Indeed, protective factors are observed by several authors and highlight the importance of the relationship issues between adults and adolescents. Some work showed that the support from adults, especially parents, and the intensity of relationships between adults and young people have an effect on protection with regard to substance use. These relationships alone cannot explain problems related to addiction, but they must be taken into account when intervening in the lives of adolescents, regardless of the nature of the intervention.

Feelings of well-being seem to be another protection factor among young people. This sense of well-being is related to living conditions and socioeconomic inequalities, which can go beyond the strict framework of addiction prevention. Interventions and prevention must be correlated with public policies to combat inequalities in order to enhance the well-being of young people. We have observed the importance of prevention messages in media campaigns, for example, which have had a positive impact on levels of experimentation with psychoactive substances.

**FIGURE 2:**
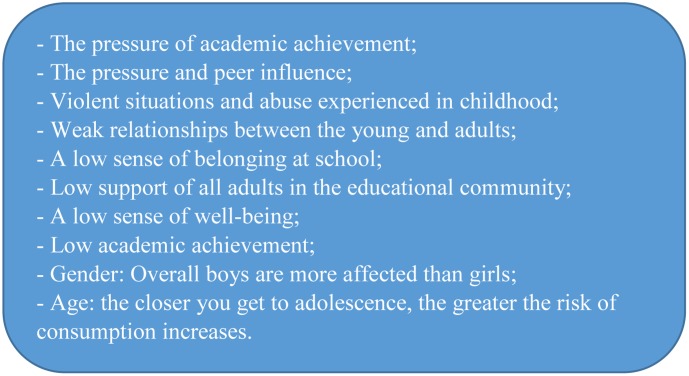
The risks factors associated with addictive behavior.

Risk and predictive factors are predominant in 12 articles on the study of the factors related to substance use. These factors are primarily environmental and are therefore not specific to children and adolescents (see **Figure 2**).

The most important risk factors studied are the stressful situations that children and adolescents face. This stress has an impact on the decisions that young people will have to make when they encounter substances. Among the many stressful situations that young people face, those that have the greatest impact are generated by their relationships with their peers. Aggressive and violent situations (Ridenour et al., 2012; Liu et al., 2013), peer pressure, and peer influence (Donovan et al., 1991; Gaffar et al., 2013) have a significant impact on young people’s substance consumption.

Our review also showed that there are many different prevention programs that could be effective for reducing consumption and children and adolescents’ addictive behavior. Some common elements highlight several effective components. First, all of these programs included a certain number of interventions that were carried out over a relatively long period. The number and the average duration of the interventions is difficult to determine due to a lack of precision in the studies. However, all interventions consisted of sessions incorporated into young people’s schedules, particularly during school time. More than one intervention is necessary in order for the program to achieve the overall objective of reducing or preventing young people’s consumption. One intervention is not enough. A “one-time” intervention can, for example, meet the objective of giving information on a topic, but it will not meet a target of prevention (Medina-Mora, 2005). Therefore, time is a major factor in the effectiveness of a program. There are no interventions with fewer than 10 sessions (and at least 45 min per session) in all of the programs included in the 12 articles. In some studies, the duration of the interventions is at least two school years and the total time during which the sessions are held is less than 3 months. Finally, the training of stakeholders in the implementation of the prevention program (teachers, parents, and social workers) is another important element.

Concerning the population, few studies specifically focused on children aged 6 to 11, while the majority focused on the 10–14 age group. These studies deal mostly with first-time experimentation with substances, in particular with alcohol, tobacco, and cannabis. It should be noted that adolescence is also a period which is associated with risk taking (Duncan et al., 1997). Understandably, it attracts the attention of those adults connected with the teenagers, and is a young people’s health public policy issue. However, some corpus studies have shown the positive effect of acting sooner in order to empower children to cope with situations and therefore delay experimentation for as long as possible. For some authors it is important to develop strategies for more targeted preventive interventions in children and adolescents who have already passed the experimentation stage.

In the corpus we also looked at emerging studies on new addictions to the Internet, smartphones, and video games. But if the prevention of legal and illegal drug use has been the subject of many studies among the teenage population for 30 years (Kandel and Logan, 1984; Botvin et al., 1995; Chen and Kandel, 1995; Duncan et al., 1997; Botvin and Kantor, 2000), the prevention of behavioral addictions, such as Internet or video game addictions, is just beginning (Sussman, 2012). Moreover, the development of fast and recent Internet-related technologies, such as smartphones and tablets, affects a significant number of young people and characterizes a whole generation of “digital natives” (Bennett et al., 2008). This data is important for prevention and is also discussed in terms of care. For example, disorders related to the use of games on the Internet have been present since 2013 in DSM-V (p. 795), (American Psychiatric Association, 2013).

Most of the studies were conducted in a school context. This over-representation of the school environment can be explained by different points related to the context: the presence of a captive audience, the possibility of applying it equally to the greatest number of people, its credibility, and the importance of the environment in children’s lives (James, 2011; Huang et al., 2013). However, other living environments, such as the socio-educational environment, community sports and of course the family environment, should not be neglected. Sixteen articles of the corpus studies have shown the importance of a joint intervention with adults who are not from the school, such as parents, educators, and social workers. An ecosystem approach is therefore considered appropriate by several authors, as it ensures the positive effects of the interventions in terms of lowering consumption and delaying the age of experimentation, among other things (Collins et al., 2011; Inman et al., 2011; O’neill et al., 2011; Lan and Lee, 2013; Spoth et al., 2013).

We have observed that in all of the articles of this review, neither health care professionals nor addiction experts are present in the preventive interventions with children. As we reported, the school context is over-represented in the 32 articles of the corpus and it is therefore unsurprising that it is the teachers who are most often requested to intervene. Other professionals, such as social workers and psychologists, are also represented, although to a lesser extent. Parents are asked to participate in all of this review’s studies, and their active presence is a necessity when trying to engage effectively with children. This data validates the legitimacy of adults with an educational mission (parents, teachers, educators, social workers) (Duff et al., 2003) to intervene with younger children.

This leads us to consider new studies and interventions in different living environments for children, and to envision an ecosystem approach, (McLeroy et al., 1988; Stewart-Brown, 2004) which would be appropriate when considering addiction prevention in children aged 6 to 11 years old.

**FIGURE 3:**
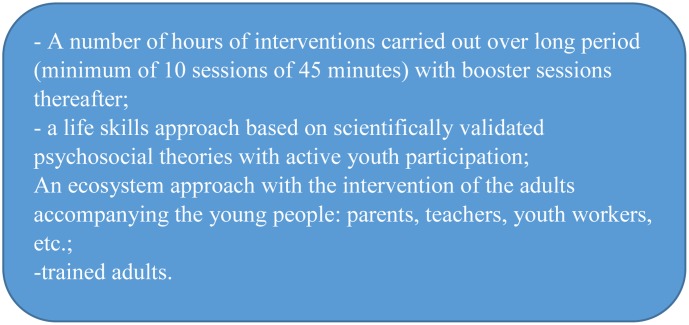
Keys elements of an effective intervention.

For several decades, research has shown what effective interventions in addiction prevention among young people is (see **Figure 3**). Currently, the issue is particularly focused on implementations already used in studies, and the opportunities for interventions to be truly implementable in different and varied contexts (Ellickson, 2014), especially in randomized controlled trials. According to Mukoma and Flisher (2004) the complexity of the field of promoting youth health, which includes addiction prevention, means that making use of randomized controlled trials is inefficient when it comes to determining the conditions of transferability and implementation of interventions known to be effective. Methodological efforts are there to provide a better understanding of this issue. According to Özdemir and Giannotta (2014) a research path that should be prioritized, concerns the study of processes which would allow us to understand the operation of a program and in particular how to implement it successfully. In order to do this, the research should be carried out by associated field stakeholders who are recognized as experts in the description and the function of the implementation context, as well as researchers. In this respect, the methods based on the model “Holistic Assessment Systems” should be considered. As the motivation of the person involved is the primary determinant of how effective the implementation is, new questions emerge. How should we negotiate the intrinsic subjectivity of the subjects’ motivation? Is effective intervention then linked specifically to the opportunities seized by the players? If we consider this type of activity as belonging to a systemic approach (Hawe and Potvin, 2009), should we, ultimately, take into account an intervention which is assessed as being efficient when it will only have a limited impact? Or should we rather accept a longer-lasting work, which can permanently change the living environment of children and adolescents and which would go beyond the scope of a program?

The intervention of adults who work together is considered to be effective. So far, we have little knowledge and few models on the logic of collaborative work on topics related to children’s health. The interest is therefore to understand these situations by bringing together researchers and practitioners; it is the purpose of intervention research (Hawe and Potvin, 2009).

Despite the amount of information observed by this review, some limitations must be addressed. First, the choice to use a bio psycho social model and so the use of three different databases meant that we had different types of papers, with different assessments, theoretical backgrounds, etc. These differences made categorization difficult. At the same time, it is possible that some relevant articles were not identified as they were not included in searched databases. In fact, since the current review includes empirical articles published in “English-speaking” journals, it is possible that studies published in other languages were excluded *a priori* from the electronic search (moreover, ISI WoS and Scopus databases include many articles in other language than English). Another limitation was that we did not focus our research strategies only on the assessment of prevention programs. So this review cited other types of articles as well as prevalence articles, but this part is probably not comprehensive. Moreover, the choice to select only peer-reviewed journals exclude very recently advanced (or submitted) articles in this field which could be under evaluation.

Finally, the choice to focus this review on addictive behaviors may have prevented us from detailing innovative and effective but more generalist programs. For example, a recent field which is of great interest are programs based on positive psychology (see for a recent review Shankland and Rosset, 2016).

## Conclusion

Our literature review allowed us to assess the current knowledge on the prevention of addictions with children and adolescents. Risk and protection factors have been studied in relation to addictions, and the study of effective prevention programs has enables us to make a few recommendations for stakeholders in the field. Before considering the multifactorial aspects of experimentation and the start of addiction, one of the major challenges over the coming years will be to create a multidisciplinary dynamic in different fields of research comprising educationalists and prevention professionals. These research and field practices will allow us to build effective, integrated programs that can be implemented in many contexts. This review also highlights the heterogeneity of goals and assessments of prevention programs, and the need for common tools in order to be able to draw better conclusions on their effectiveness.

## Author Contributions

All authors listed have made a substantial, direct and intellectual contribution to the work, and approved it for publication.

## Conflict of Interest Statement

The authors declare that the research was conducted in the absence of any commercial or financial relationships that could be construed as a potential conflict of interest.
